# Delineation of the Germline and Somatic Mutation Interaction Landscape in Triple-Negative and Non-Triple-Negative Breast Cancer

**DOI:** 10.1155/2020/2641370

**Published:** 2020-07-06

**Authors:** Jiande Wu, Tarun K. K. Mamidi, Lu Zhang, Chindo Hicks

**Affiliations:** ^1^Department of Genetics, Louisiana State University Health Sciences Center, School of Medicine, New Orleans, LA 70112, USA; ^2^Graduate Biomedical Sciences, The University of Alabama at Birmingham, Birmingham, AL 35233, USA; ^3^Department of Public Health Sciences, Clemson University, Clemson, SC 29634, USA

## Abstract

**Background:**

Breast cancer development and progression involve both germline and somatic mutations. High-throughput genotyping and next-generation sequencing technologies have enabled discovery of genetic risk variants and acquired somatic mutations driving the disease. However, the possible oncogenic interactions between germline genetic risk variants and somatic mutations in triple-negative breast cancer (TNBC) and non-triple-negative breast cancer (non-TNBC) have not been characterized. Here, we delineated the possible oncogenic interactions between genes containing germline and somatic mutations in TNBC and non-TNBC and investigated whether there are differences in gene expression and mutation burden between the two types of breast cancer.

**Methods:**

We addressed this problem by integrating germline mutation information from genome-wide association studies with somatic mutation information from next-generation sequencing using gene expression data as the intermediated phenotype. We performed network and pathway analyses to discover molecular networks and signalling pathways enriched for germline and somatic mutations.

**Results:**

The investigation revealed signatures of differentially expressed and differentially somatic mutated genes between TNBC and non-TNBC. Network and pathway analyses revealed functionally related genes interacting in gene regulatory networks and multiple signalling pathways enriched for germline and somatic mutations for each type of breast cancer. Among the signalling pathways discovered included the DNA repair and Androgen and ATM signalling pathways for TNBC and the DNA damage response, molecular mechanisms of cancer, and ATM and GP6 signalling pathways for non-TNBC.

**Conclusions:**

The results show that integrative genomics is a powerful approach for delineating oncogenic interactions between genes containing germline and genes containing somatic mutations in TNBC and non-TNBC and establishes putative functional bridges between genetic and somatic alterations and the pathways they control in the two types of breast cancer.

## 1. Introduction

Breast cancer is the second most diagnosed malignancy and the second leading cause of cancer-related deaths among women in the US [1]. In 2018, there were 266,120 new cases of breast cancer diagnosed among women and 40,920 women died from the disease in US [1]. Breast cancer is a heterogeneous disease comprising of two types, triple-negative breast cancer (TNBC) and non-triple-negative breast cancer (non-TNBC) [2, 3]. The majority of breast cancers are non-TNBC [2, 3]. These cancers are characterized by less aggressive clinical behaviour; respond to targeted, endocrine, and human epidermal growth factor receptor- (HER2-) directed therapies; and generally have a good prognosis and good clinical outcomes [2, 3]. The TNBC types are defined as breast cancers that lack expression of the oestrogen receptor (ER) and progesterone receptor (PR) and lack amplification of the human epidermal growth factor receptor 2 (HER-2) [4–6]. Unlike the non-TNBC type of breast cancers, TNBC tumors belong to the biologically aggressive type of breast cancer and cannot be managed with targeted, endocrine, or HER2/neu-directed therapies [4–6]. Cytotoxic chemotherapy remains the only effective therapeutic modality for TNBC. This study focuses on TNBC and non-TNBC because both clinical and epidemiological studies have shown that TNBC has a poor outcome and survival rate when compared to non-TNBC [7–12].

Breast cancer development is driven by acquired driver somatic mutations; however, germline genetic variants play a role in tumorigenesis by partaking in critical biological and cellular processes. For decades, germline mutations, contained within the heritable genome, and somatic mutations, acquired de novo by breast cancer cells, have been considered as separate research endeavours, and each has unique clinical applications and implications for patient care. A critical challenge faced by clinicians and patients is the identification of patients at high risk of developing aggressive disease that could guide the application of precision medicine and precision prevention in TNBC and non-TNBC. Achieving that goal requires understanding the germline somatic mutation interaction landscape and discovery of molecular markers driving each disease and distinguishing the two types of breast cancer.

Advances in microarray technology have enabled molecular classification of TNBC and non-TNBC [13, 14]. At least two of these signatures, the Prosigna (PAM50), a 50-gene signature, and MammaPrint, a 70-gene signature, have proven to be useful for prognostic purposes in the clinic [15–19]. However, although these primary analyses have enabled deciphering the molecular taxonomy of breast cancer and discovery of prognostic markers, they have been unsuccessful in determining which genes have causative roles in the two types of breast cancer as opposed to being consequences of the disease states [20]. High-throughput genotyping and reduction in genotyping costs have enabled discovery of genetic variants associated with an increased risk of developing breast cancer using genome-wide association studies (GWAS) [21–24]. These discoveries have opened new options in clinical assessment of the risk of developing breast cancer [21–24]. For example, genetic variants from GWAS are now being incorporated in risk prediction models such as polygenic risk scores for predicting the risk of developing breast cancer and breast cancer subtypes [25, 26]. However, despite this remarkable progress, the causal association between genetic susceptibility and tumorigenesis in the two types of breast cancer has not been completely characterized.

The recent surge of next-generation sequencing of the cancer genomes has opened new options in clinical oncology, from discovery of driver mutations to implementation of precision medicine [27, 28]. Comprehensive catalogues of somatic mutations have been developed by large multicenter and multinational projects such as The Cancer Genome Atlas (TCGA) and the International Cancer Genetics Consortium (ICGC) [28, 29]. However, the full breadth of the goals of the TCGA and the ICGC projects is running into several bottlenecks in translating the findings into clinical practice to improve human health. One of the more significant bottlenecks is the inability to integrate germline mutation with somatic mutation information to delineate the germline-somatic mutation interaction landscape in TNBC and non-TNBC and to discover genetically altered features distinguishing the two types of breast cancer. Given the emerging evidence indicating that germline mutations may interact with somatic events to drive carcinogenesis [30–33], integrating germline and somatic mutation information holds promise not only for causally associating genetic susceptibility with tumorigenesis but also for defining the molecular networks and signalling pathways through which they interact and cooperate.

The objective of this study was to delineate germline and somatic mutation interaction landscape in TNBC and non-TNBC and to determine whether there are differences in gene expression and somatic mutation burden between the two types of breast cancer. We focus on the two types of breast cancer. Our working hypotheses were that (1) genomic alterations in genes containing germline and somatic variations could lead to measurable changes associating genetic predisposition with tumorigenesis and distinguishing TNBC from non-TNBC and (2) integrative analysis combining germline and somatic mutation information at the gene level will uncover molecular networks and signalling pathways through which germline and somatic variations interact and cooperate to drive TNBC and non-TNBC. We addressed these hypotheses using an integrative genomic approach that integrates germline variation information from GWAS with somatic mutation information from next-generation sequencing on TNBC and non-TNBC from TCGA, using gene expression data from TCGA as the intermediate phenotype. Our modelling approach focuses on the genes, gene regulatory networks, and signalling pathways rather than on individual mutations. This robust approach was designed to establish the potential causal association between genetic predisposition and tumorigenesis and to provide valuable insights about the broader biological context in which germline and somatic mutations interact and cooperate to drive TNBC and non-TNBC. It is worth noting that the two subtypes of breast cancer have many subtypes which we did not consider here, a weakness that we readily acknowledge as it is beyond the scope of this investigation. As pointed out earlier in this section, our focus on TNBC and non-TNBC was motivated by evidence from both clinical and epidemiological studies showing that TNBC has poorer outcomes and poorer survival rates when compared to non-TNBC [7–12].

## 2. Material and Methods

**Figure 1:**
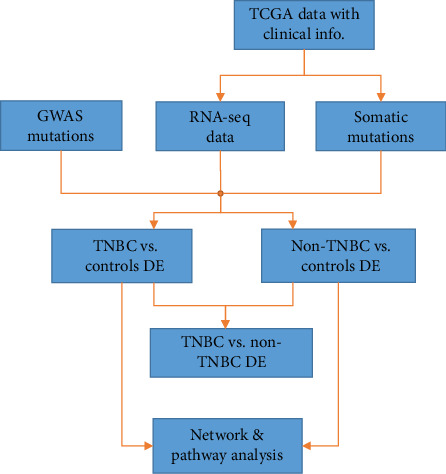
Project design, data processing, and analysis workflow for integrative analysis combining germline with somatic mutation information in TNBC and non-TNBC using gene expression data as the intermediate phenotype. RNA-seq read count data and somatic information were downloaded from the TCGA via the GDC. Germline mutation information was manually curated from GWAS studies and supplemented with information from the GWAS catalogue. LIMMA (R) package was used for the discovery of differentially expressed (DE) mutated and nonmutated genes. Ingenuity Pathway Analysis (IPA) was used for the discovery of molecular networks and biological pathways enriched for germline and somatic mutations.

Advances in high-throughput genotyping and next-generation sequencing technologies enabled discovery and creation of comprehensive catalogues of germline and somatic mutations. These discoveries have increased our understanding of the genetic susceptibility landscape and the molecular taxonomy of breast cancer. However, analyses of germline and somatic mutations have historically been considered as separate endeavours in breast cancer research. With the availability of germline, somatic, and gene expression variation data and powerful bioinformatics tools, we are now well-positioned to understand the causal association between genetic susceptibility and tumorigenesis through integrative analysis. Here, we integrated data on germline, somatic, and gene expression variation to delineate the germline-somatic mutation interaction landscape in TNBC and non-TNBC. The overall study design and execution strategy used in this study is presented in Figure 1. Below, we provide a detailed description of the sources of germline, somatic, and gene expression gene expression variation data along with clinical data used in this investigation, as well as the data processing and analysis strategies used.

### 2.1. Germline Mutations and Associated Genes

We used population-level GWAS discoveries, specifically single-nucleotide polymorphisms (SNPs) (herein referred to as germline mutations) and genes associated with an increased risk of developing breast cancer from a comprehensive catalogue that we have developed and published [20, 21] and recently updated [34, 35]. The catalogue was created by manually extracting, curating, and annotating germline mutations and genes from published GWAS reports using the guidelines proposed by the Human Genome Epidemiology Network for Systematic Review of Genetic Associations [36–40]. The information in our catalogue was supplemented with information from the GWAS catalogue which is continuously updated, to ensure completeness of the germline variation data used in this study [22–24]. The resulting data set included 754 genes and their chromosome positions, SNPs and their identification numbers (rs-IDs), and evidence of association as determined by the GWAS *P* value as well as original published GWAS reports from which the information was derived. A complete list of genes and germline variants along with original sources of published GWAS reports from which germline mutations were derived is presented in Supplementary Table SG provided as supplementary data to this report.

### 2.2. Somatic Mutation Information and Gene Expression Data

Somatic mutation and gene expression along with clinical information were obtained from TCGA via the Genomics Data Commons (GDC) using the data transfer tool https://gdc.cancer.gov/ [41]. Somatic mutation information and gene expression data were generated on the same patient population. Gene expression was generated using RNA sequencing. Using the clinical information provided by the TCGA, we characterized TNBC as breast cancers lacking expression of the oestrogen receptor (ER), progesterone receptor (PR), and human epidermal growth factor receptor 2 (HER2) amplification. Cancers not meeting this histology-based classification were classified as non-TNBC. The original data set included 1,108 tumor samples and 113 normal control samples. We matched the somatic mutation information with gene expression data using the clinical information provided by the TCGA to identify samples with both somatic mutation and gene expression data. After this data processing step, the resulting data set consisted of *N* = 883 non-TNBC samples and *N* = 99 TNBC samples used in this study. Samples without clinical or mutation information (*N* = 126) were not included in the analysis as they could not be ascertained for mutation status and/or correctly assigned to either type of breast cancer. The data was further processed and checked for quality. We performed noise reduction by filtering or removing rows with missing data as determined by the number of reads, such that each row had at least ≥30% data points. Data filtering was performed using counts per million (CPM) filter (>0.5) implemented in the R Package [42]. Following data processing and filtering, we normalized the resulting data set using the trimmed mean of *M* values (TMM) normalization method and log transformed the data using the Voom module in the LIMMA package implemented in R [42]. Processed and normalized data contained 36,451 probes and was used for downstream analyses. Prior to analysis, the probe IDs and gene symbols and names were matched for interpretation using the Ensemble database, a database used for gene annotation in sequencing experiments and on sequencing technology platforms.

### 2.3. Data Analysis

The data processing and analysis steps are shown in the project design and execution workflow presented in Figure 1. As a first step, we performed whole transcriptome analysis comparing gene expression levels between patients diagnosed with TNBC and controls and between patients diagnosed with non-TNBC and control samples, as well as between the two types of breast cancer using the LIMMA package implemented in R [42]. This unbiased approach was designed to identify significantly differentially expressed mutated (both germline and somatic mutated) and nonmutated genes associated with each type and distinguish the two types of breast cancer. For each analysis, we used the false discovery rate (FDR) procedure to correct for multiple hypothesis testing [43].

The genes were ranked on *P* values and FDR. Significantly differentially expressed genes in each type and between the two types of breast cancer were grouped as either mutated or not mutated. Somatic mutated genes were further assessed for the number of mutation events per gene within each type and in both types of breast cancer to discover differentially mutated genes between TNBC and non-TNBC. A gene was considered highly mutated if the number of mutation events was ≥3. A gene was considered differentially mutated if it was only mutated in one type of breast cancer. Significantly differentially expressed genes without mutations were grouped into four groups, genes significantly associated with TNBC, genes significantly associated with non-TNBC, genes significantly associated with both diseases, and genes distinguishing the two diseases.

To discover significantly differentially expressed and differentially somatic mutated genes distinguishing TNBC from non-TNBC, we compared gene expression levels and number of mutation events per gene between the two types of breast cancer. Genes associated with both types of breast cancer were not included in this analysis to avoid confounding of the results. Differentially somatic mutated genes were identified by counting the number of mutation events per gene in each type of breast cancer. If the gene had somatic mutations in only one type of breast cancer, it was considered differentially mutated. To identify genes containing germline and somatic mutations, we evaluated all the 754 genes containing germline mutations for the presence of somatic mutations and their association with each type of breast cancer measured by their expression. Germline mutated genes significantly associated with each type of breast cancer were further evaluated for differences in their expression levels and somatic mutations between the two types of breast cancer.

We used the Core Analysis and pathways build modules implemented in the Ingenuity Pathway Analysis (IPA) software platform, QIAGEN Inc., USA [44], to model the gene regulatory networks and signalling pathways enriched for germline and somatic mutations. To characterize the mutated genes according to the biological processes, molecular functions, and cellular components in which they are involved, we used the Gene Ontology (GO) database as implemented in IPA [45]. We performed network and pathway analyses separately for TNBC and non-TNBC. For each analysis, we mapped highly significantly differentially expressed genes containing both germline and somatic mutations and highly somatic mutated genes without germline mutations but were highly significantly associated with each type of breast cancer onto networks and canonical pathways. IPA assigned genes to molecular functions, networks, and the signalling pathways they are involved. Generated networks and pathways were ordered by *Z* score and *P* values (log *P* values), respectively; indicating the level of significance for correctly assigning the mutated genes to the network, functional category, and pathways. Significance of molecular functions and the canonical pathways was tested by the Fisher exact test as implemented in IPA. To ensure the reliability of the predicted networks, we used the trim module implemented in IPA to filter out networks with ≤3 connections and genes without any connections.

### 2.4. *In Silico* Validation and Assessment of Potential Clinical Utility

To test whether the genes containing germline and somatic mutations discovered in this investigation have clinical utility and to validate them as potential clinically actionable biomarkers, we evaluated them against two clinically validated assays as described below:

1. For the first assay, we used the Prosigna (PAM50), a 50-gene signature that has gained prominence in clinical applications as a prognostic gene signature in breast cancer [15–17]. The rationale for using this assay is based on the recognition that the prognostic value of the PAM50 intrinsic gene signature has been shown to be predictive of risk of recurrence, a common feature in TNBC, and benefit of chemotherapy, the only effective therapeutic modality for TNBC [15–17]
2. For the second assay, we used the MammaPrint, a clinically validated assay consisting of 70 genes developed by Agendia Corporation [18, 19]. MammaPrint is an FDA-cleared microarray-based test that uses expression levels of the 70 MammaPrint genes to assess distant recurrence risk in early-stage breast cancer. The rationale for using this assay is based on the recognition that the MammaPrint is a prognostic tool used for predicting recurrence risk of breast cancer [18, 19]. TNBC has very high recurrence rates; thus, use of such assay to assess the potential for the risk of recurrence is justified

We chose the two assays because both the PAM50 and MammaPrint were developed using gene expression, which is also used in this investigation as the intermediate phenotype. For these validation analyses, we used several approaches: First, we investigated whether the genes containing both germline and somatic mutations are present in the PAM50 and the MammaPrint assays. Second, we evaluated the genes in these assays against highly somatic mutated genes significantly associated with each disease to eliminate the bias imposed by the limited number of genes containing germline mutations. Third, we investigated whether the genes containing germline and/or somatic mutations significantly associated with each disease are functionally related and interact with genes in the PAM50 and/or MammaPrint assays. The third approach was necessitated by the limited number of the genes in each assay. We reasoned that genes in these clinically validated assays may be regulated or may be regulating other genes which are altered in the germline, somatic, or both genomes.

## 3. Results

### 3.1. Discovery of Somatic Mutated and Nonmutated Gene Signatures

We compared gene expression levels between TNBC and controls and between non-TNBC and controls to discover and characterize signatures of mutated and nonmutated genes associated with the two types of breast cancer. Genes were ranked and selected using estimates of *P* values adjusted for multiple hypothesis testing. Comparison of gene expression levels between patients with TNBC and controls produced a signature of 22,968 significantly differentially expressed genes (*P* < 0.05), of which 5,502 genes contained somatic mutations and 17,466 genes had no somatic mutations. Comparison of gene expression levels between patients with non-TNBC and controls produced a signature of 19,463 significantly differentially expressed genes (*P* < 0.05), of which 11,399 genes contained somatic mutations and 8,064 genes were without somatic mutations. A complete list of somatic mutated genes significantly associated with TNBC and non-TNBC is presented in Supplementary Table SM. A complete list of genes without somatic mutations significantly associated with TNBC and non-TNBC is presented in Table SN.

**Figure 2:**
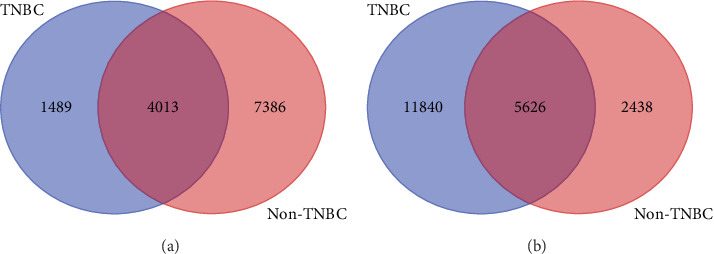
Venn diagrams showing the distribution of genes containing somatic mutations (a) and genes without somatic mutations (b) significantly differentially expressed between cases and control samples in TNBC and non-TNBC. Genes in the intersections were significantly associated with both types of breast cancer.

To discover gene signatures uniquely associated with each type of breast cancer and gene signatures associated with both types of breast cancer, we evaluated mutated and nonmutated genes using adjusted *P* values derived from analysis of gene expression. A summary of the results showing the distribution of mutated and nonmutated genes significantly associated with each type and both types of breast cancer is presented in Venn diagrams in Figure 2. In each figure, the number of genes significantly associated with both types of breast cancer is shown in the intersection of the Venn diagram.  Figure 2(a) presents genes containing somatic mutations and are significantly associated with each type or both types of breast cancer.  Figure 2(b) presents genes without somatic mutations significantly associated with each type or both types of breast cancer.

Among the somatic mutated genes (Figure 2(a)), 1,489 genes were significantly associated with TNBC and 7,386 genes were significantly associated with non-TNBC, whereas 4,013 were significantly associated with both types of breast cancer. Among the genes without somatic mutations (Figure 2(b)) 11,840 genes were significantly associated with TNBC and 2,348 genes were significantly associated with non-TNBC, whereas 5,626 genes were significantly associated with both diseases. A complete list of somatic mutated genes significantly associated with TNBC is presented in Supplementary Table S2A1. A complete list of somatic mutated genes significantly associated with non-TNBC is presented in Supplementary Table S2A2. These analyses confirmed our hypothesis that genomic alterations in genes containing somatic mutations could lead to measurable changes associating them with TNBC, non-TNBC, or both. Overall, the analysis showed that both somatic mutated and nonmutated genes are associated with each type of breast cancer and that some mutated and nonmutated genes tend to affect both types of breast cancer.

### 3.2. Differentially Expressed and Differentially Mutated Gene Signatures

Having discovered signatures of mutated and nonmutated genes associated with each type and/or both types of breast cancer, we performed additional analysis to investigate the differences in gene expression and mutation burden between TNBC and non-TNBC. For this analysis, we created and analysed a new data set of 8,875 genes, which was generated by combining the 1,489 genes containing somatic mutations significantly associated with TNBC only and the 7,386 genes containing somatic mutations significantly associated with non-TNBC only. Genes associated with both types of breast cancer were not included in this analysis to eliminate confounding of the results.

The analysis revealed a signature of 6,887 significantly differentially expressed genes distinguishing TNBC from non-TNBC. The signature included 290 genes somatic mutated in TNBC, 4,957 genes somatic mutated in non-TNBC, and 1,640 genes somatic mutated in both types of breast cancer. A list of the top 30 highly significantly differentially expressed somatic mutated genes between TNBC and non-TNBC with high somatic mutation events per gene is presented in Table 1. A complete list of genes significantly differentially expressed and mutated between the two types of breast cancer is presented in Supplementary Table S1. Also presented in Table S1 are significantly differentially expressed genes with somatic mutations in both types of breast cancer.

This confirmed our hypothesis that there are differences in gene expression and somatic mutation burden between TNBC and non-TNBC. Additionally, the results showed that some of the differentially expressed genes tend to be somatic mutated in both types of breast cancer. Overall, there was significant variation in the number of somatic mutations per gene for genes mutated in each type and/or both types of breast cancer. The number of somatic mutation events per gene for the genes mutated in TNBC ranged from 1 to 3. The most highly mutated genes were *COPE*, *ENPP5*, and *RBM22* (Table 1). For genes mutated in non-TNBC, the number of somatic mutation events per gene ranged from 1 to 99. The most highly mutated genes were *GATA3*, *FOXA1*, *FRMPD4*, and *WNK3* (Table 1). Interestingly, genes associated with non-TNBC had higher somatic mutation events per gene than genes associated with non-TNBC (Table 1). The number of somatic mutation events per gene was not evenly distributed for the genes mutated in both types of breast cancer. The results confirmed our hypothesis that for selected set of genes, there are significant differences in mutation burden and gene expression levels between TNBC and non-TNBC, suggesting that the two types of breast cancer may be amenable to mutation-based classification.

### 3.3. Discovery of Germline and Somatic Mutated Gene Signatures

As noted earlier in Introduction, breast cancer develops through somatic driver mutations; however, germline mutations can potentiate tumorigenesis via diverse mechanisms. To establish the association between germline and somatic mutation information, we performed additional analysis. We hypothesized that genes containing germline mutations also contain somatic mutations and that these genes are associated with either TNBC or non-TNBC or both. As the first step in addressing this hypothesis, we evaluated all the 754 genes containing germline mutations associated with an increased risk of developing breast cancer for association with each type or both types of breast cancer using gene expression *P* values and somatic mutation information. Out of the 754 genes with germline mutations, 632 genes matched the probes in the TNBC data set and 611 genes matched the non-TNBC data set and were used in the evaluation. The small discrepancy between the original set of genes and the resulting two subdata sets was due to annotation and filtering as described in Material and Methods.

**Figure 3:**
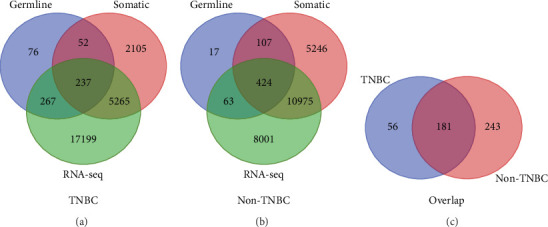
Venn diagram showing the distribution of genes containing both germline and somatic mutations, germline mutations only, and somatic mutations only and nonmutated in (a) TNBC and (b) non-TNBC. (c) Venn diagram showing the overlap in genes containing both germline and somatic mutations in TNBC and non-TNBC.

The results showing the distribution of germline and somatic mutated genes and nonmutated genes from these analyses are presented in Venn diagrams in Figure 3 for each type and both types of breast cancer. For TNBC, we discovered 289 genes containing both germline and somatic mutations (Figure 3(a)). A subset of these genes, 237 genes, was significantly associated with TNBC (Figure 3(a)). In addition, 267 genes containing germline mutations only were significantly associated with TNBC (Figure 3(a)). The remaining 76 germline mutated genes did not contain somatic mutations and were not associated with the disease. Supplementary Table SA3 presents a complete list of germline mutated genes with or without somatic mutations significantly associated with TNBC.

When we evaluated germline mutated genes for the presence of somatic mutations and association with non-TNBC, we discovered 531 genes containing both germline and somatic mutations (Figure 3(b)). A subset of these genes, 424 genes, was significantly associated with non-TNBC (Figure 3(b)). The analysis also revealed 63 genes containing germline mutations only significantly associated with the disease (Figure 3(b)). The remaining 17 germline mutated genes did not contain somatic mutations and were not associated with the disease (Figure 3(b)). A complete list of all germline mutated genes with or without somatic mutations significantly associated with non-TNBC is presented in Supplementary Table SB3.

Following the discovery of genes containing both germline and somatic mutations associated with each type and both types of breast cancer, we performed additional evaluation to discover genes containing both germline and somatic mutations uniquely associated with TNBC and non-TNBC or both. This evaluation was restricted to 661 genes (i.e., 237 genes containing both germline and somatic mutations associated with TNBC plus 424 genes containing both germline and somatic mutations associated with non-TNBC). The results of this evaluation are presented in Figure 3(c). We discovered 56 genes containing both germline and somatic mutations uniquely associated with TNBC, 243 genes containing both germline and somatic mutations uniquely associated with non-TNBC, and 181 genes containing both germline and somatic mutations associated with both types of breast cancer (Figure 3(c)).

Having discovered gene signatures enriched for germline and somatic mutations associated with each type of breast cancer, we evaluated the genes in the signatures for the number of mutation events per gene, focusing on genes containing both germline and somatic mutations and associated with each type of breast cancer. The results showing a list of the top 30 highly somatic mutated genes out of the 237 genes containing both germline and somatic mutations associated with TNBC are presented in Table 2(a). The list included the genes *ARID1B*, *BRCA1*, *ERBB4*, *ARHGAP5*, *EFR3B*, *AKAP9*, *ASH1L*, *ATM*, *BAHCC1*, and *HAST9* containing germline mutations reported to be directly associated with TNBC (Supplementary Table SG) and the genes *MSH3*, *RELN*, and *MYO10* containing genetic variants weakly associated with TNBC. The analysis also revealed the genes involved in DNA repair including *BRCA1*, *ATM*, and *MSH3* strongly associated with TNBC. A complete list of all the genes containing both germline and somatic mutations significantly associated with TNBC is presented in Supplementary Table S3A.

The results showing a list of the top 30 most highly somatic mutated genes out of the 424 genes containing both germline and somatic mutations associated with non-TNBC are presented Table 2(b). The genetic predisposing genes discovered included the genes *RB1*, *ATA*, *ERBB2*, and *ATR* containing germline mutations with small effects (Table 2(b)). A complete list of all the genes containing both germline and somatic mutations significantly associated with non-TNBC is presented in Supplementary Table S3B. There was an overlap in genes containing both germline and somatic mutations with some genes significantly associated with each type of breast cancer showing mutations in both types of breast cancer.

To address the hypothesis that the 56 genes containing both germline and somatic mutations uniquely associated with TNBC and the 243 genes uniquely associated with non-TNBC (Figure 3(c)) are significantly differentially expressed and differentially mutated between the two types of breast cancer, we compared their expression levels and number of mutation events. The analysis revealed a signature of 251 significantly differentially expressed genes containing both germline and somatic mutations distinguishing two types of breast cancer. Among them, 7 genes were somatic mutated in TNBC, 181 genes were somatic mutated in non-TNBC, and 63 genes were somatic mutated in both types of breast cancer.  Table 2(c) shows a list of the top 30 significantly differentially expressed and differentially mutated genes between TNBC and non-TNBC. A complete list of all the 251 genes containing both germline and somatic mutations significantly differentially expressed distinguishing TNBC from non-TNBC is presented in Supplementary Table S3C.

The most highly mutated genes in TNBC were *AGPAT9*, *FKBPL*, *IGSF21*, *BABAM1*, *MCM8*, *MYC*, and *NFIX*. The most mutated genes in non-TNBC were *FRMD4A*, *KCNH7*, *ABCC4*, *CCDC88C*, *CDH12*, *MAGI3*, *TNS1*, *BLM*, *DNM3*, *L3MBTL3*, *BRIP1*, *FOXP1*, *ABCF1*, *ADCY3*, *COL1A1*, *CUX1*, *EWSR1*, *EXOC3*, and *IGF1R* (Table 2(c)). The analysis confirmed our hypothesis that a selective set of genes containing germline and somatic mutations is differentially expressed and differentially mutated between TNBC and non-TNBC, highlighting the need for joint analysis of genotype and somatic mutation data in biomarker discovery in breast cancer.

### 3.4. Molecular Networks and Biological Pathways

To delineate the possible oncogenic interactions and cooperation between genes containing germline and somatic mutations, we performed network and pathway analyses as described in Material and Methods separately, for each type of breast cancer. For TNBC, we used the 56 genes containing both germline and somatic mutations uniquely associated with TNBC and the 99 highly somatic mutated genes (i.e., ≥5 somatic mutation events per gene) that were highly significantly associated with TNBC. Likewise, for non-TNBC, we used the 243 genes containing both germline and somatic mutations uniquely associated with the disease and the 246 highly somatic mutated associated with the disease. The rationale for including highly somatic mutated genes without germline mutations was driven by the realization that GWAS discoveries explain only a small proportion of the phenotypic variation. Crucially, genetic variants from GWAS may not necessarily be causal but may be interacting and cooperating with highly somatic mutated oncogenes involved in the causal mechanisms through *trans*-regulation. Thus, limiting the analysis to only genes containing both germline and somatic mutation could miss important driver genes, gene regulatory networks, and signalling pathways. Using this approach, we discovered multiple molecular networks and multiple signalling pathways enriched for germline and somatic mutations.

**Figure 4:**
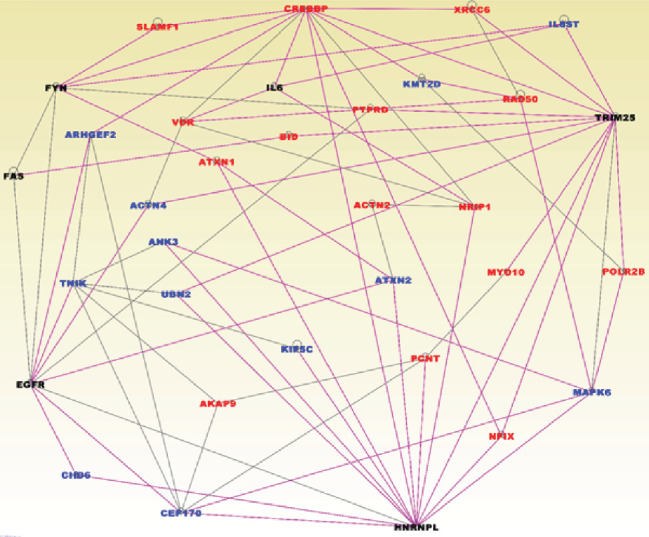
Molecular networks enriched for germline and somatic mutations in TNBC. Genes in red font contain germline and somatic mutations, and genes in blue font contain germline mutations only. Nodes represent the genes, and vertices represent functional relationships. Genes in black fonts are functionally mutated genes.

The results showing molecular networks enriched for germline and somatic mutations in TNBC are presented in Figure 4. In the figure, genes containing both germline and somatic mutations are presented in red fonts and genes containing somatic mutations only are presented in blue fonts. Network analysis revealed 12 molecular networks enriched for germline and somatic mutations. The networks contained genes with multiple overlapping functions. Among them were genes predicted to be involved in cellular function and maintenance, cellular response to therapeutics, hereditary disorder, cell cycle, cell to cell signalling, cancer, cell death, and survival (Figure 4). We discovered multiple signalling pathways enriched for germline and somatic mutations including DNA repair and Androgen and *ATM* signalling pathways, all of which have been implicated in TNBC [21]. The top upstream regulators included *CD24*, *TCF20*, *PUF60*, and *RBBP4*.

**Figure 5:**
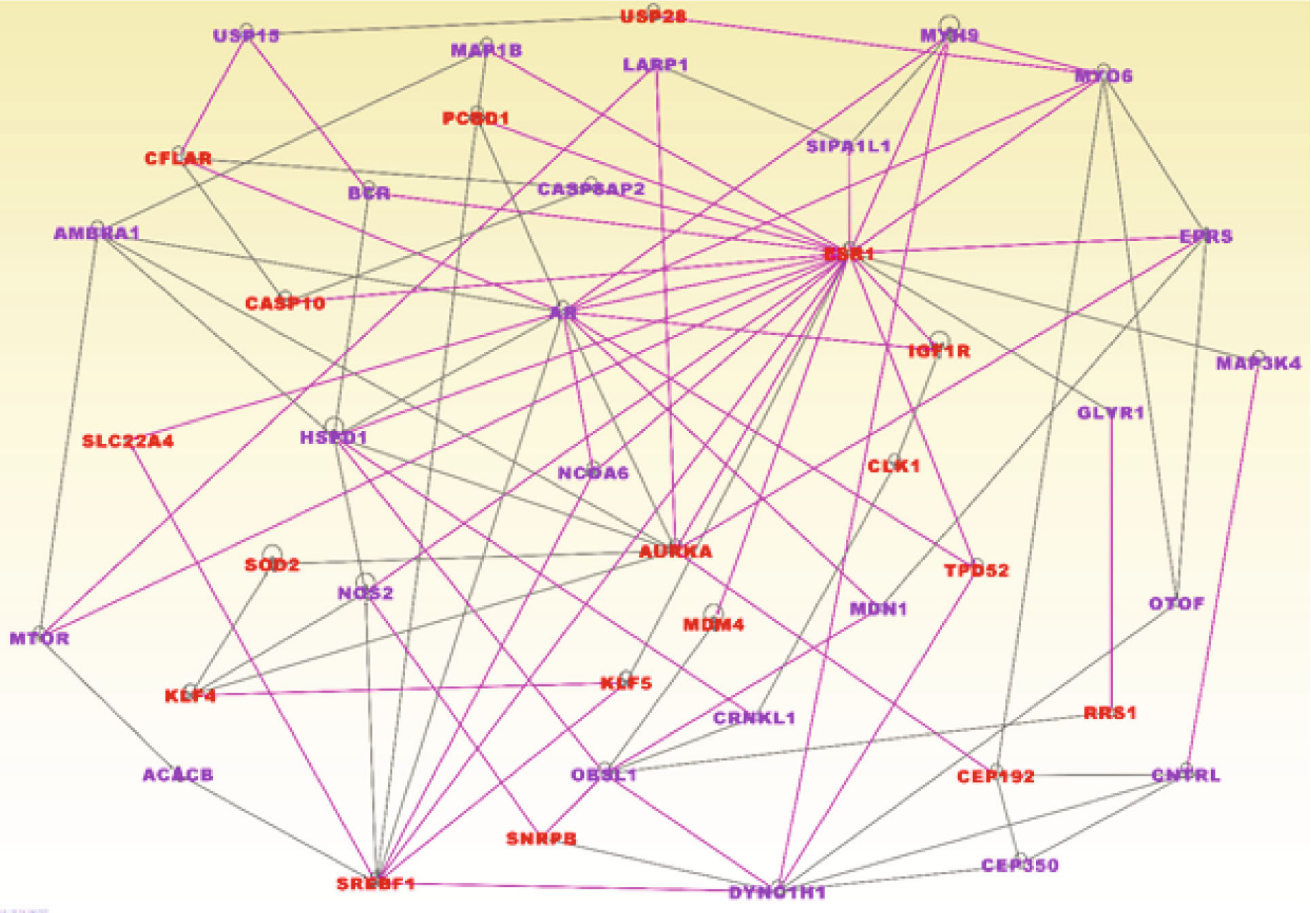
Molecular networks enriched for germline and somatic mutations in non-TNBC. Genes in red font contain germline and somatic mutations, and genes in purple fonts contain germline mutations only. Nodes represent the genes, and vertices represent functional relationships.

The results showing molecular networks enriched for germline and somatic mutations in non-TNBC are presented in Figure 5. In the figure, genes containing both germline and somatic mutations are presented in red fonts and genes containing somatic mutations only are presented in purple fonts to distinguish them from those discovered in TNBC. Network analysis revealed 25 molecular networks enriched for germline and somatic mutations (Figure 5). The networks revealed genes predicted to be involved in cancer, cellular function and maintenance, cellular response to therapeutics, hereditary disorder, cell cycle, cell to cell signalling, cancer, cell death, and survival. In addition, we discovered multiple signalling pathways enriched for germline and somatic mutations. The top pathways included hereditary breast cancer, role of *BRCA1* in DNA damage response, molecular mechanisms of cancer, and *ATM* and *GP6* signalling pathways. The top upstream regulators included *ERBB2* and *ITGB1*.

Overall, there was overlap in molecular networks and signalling pathways discovered in TNBC and non-TNBC. For example, the signalling pathways involved in DNA repair and DNA damage were discovered in both types of breast cancer. Interestingly, in both TNBC and non-TNBC, genes containing germline mutations strongly associated with breast cancer were functionally related and interacting with highly somatic mutated genes in gene regulatory networks and signalling pathways. Taken together, the results of this investigation confirmed our hypothesis that in the context of breast cancer, TNBC and non-TNBC can be considered as emergent properties of molecular networks and signalling pathways influenced by both germline and somatic mutations. The investigation revealed that integrating germline with somatic mutation information holds promise for discovering the molecular mechanisms through which germline and somatic mutations interact and cooperate to drive TNBC and non-TNBC.

### 3.5. *In Silico* Validation Using Clinically Validated Assays

To validate and investigate the potential clinical utility of the discovered germline-somatic mutated genes, we performed *in silico* validation using the two clinically validated assays as described in Material and Methods using the Prosigna (PAM50) [15–17] and the MammaPrint [18, 19]. We evaluated the 56 genes containing both germline and somatic mutations uniquely associated with TNBC and the 243 genes containing both germline and somatic mutations uniquely associated with non-TNBC against the genes in each assay separately for each type of breast cancer and obtained the following results.

Evaluation using PM50 revealed the *MYC* gene containing both germline and somatic mutations associated with TNBC. Evaluating the same assay on non-TNBC revealed four genes: *ERBB2*, *ESR1*, *PHGDH*, and *TYMS* containing both germline and somatic mutations significantly associated with that type of breast cancer. In addition, we discovered six genes: *CCNE1*, *CEP55*, *EGFR*, *EXO1*, *FGFR4*, and *MAPT* associated with both types of breast cancer. Further evaluation focusing on highly somatic mutated genes unique to TNBC revealed the genes *CDC20*, *CXXC5*, and *MYC*.

Evaluation using MammaPrint did not reveal genes containing both germline and somatic mutations significantly associated with TNBC or non-TNBC. However, the analysis revealed 3 somatic mutated genes: *CDC42BPA*, *EXT1*, and *PRC1* significantly associated with both types of breast cancer. Additionally, evaluation focusing on highly somatic mutated genes unique to non-TNBC revealed the genes *BAG1*, *BIRC5*, *BLVRA*, *CCNB1*, *CDC6*, *ERBB2*, *ESR1*, *FOXA1*, *GPR160*, *GRB7*, *KIF2C*, *KRT5*, *MELK*, *MIA*, *NAT1*, *NDC80*, *PHGDH*, *PTTG1*, *RRM2*, *SFRP1*, *SLC39A6*, *TYMS*, *UBE2C*, and *UBE2T*, confirming our hypothesis that somatic mutated genes have the promise to serve as potential clinically actionable molecular markers. Taken together, these results demonstrate that integrative analysis combining germline and somatic mutated information using gene expression as the intermediate phenotype is a powerful approach for delineating possible oncogenic interactions between germline and somatic mutations and correlating this information with clinically validated assays.

## 4. Discussion

We used an integrative genomic approach combining data on germline and somatic variation using gene expression data as the intermediate phenotype to delineate possible oncogenic interactions and cooperation between genes containing germline and somatic mutations in TNBC and non-TNBC and to investigate the difference in mutation burden between the two types of breast cancer. The investigation revealed that genes containing germline mutations also contain somatic mutations. The investigation also revealed differences in gene expression and mutation burden between TNBC and non-TNBC. Most notably, the investigation revealed multiple gene regulatory networks and signalling pathways enriched for germline and somatic mutations in each type of breast cancer. To our knowledge, this is the first study to comprehensively characterize the germline-somatic mutation interaction landscape in TNBC and non-TNBC. The link between germline and somatic mutations in breast cancer has been explored [46]. Recently, our group reported possible oncogenic interactions between genes containing germline and somatic mutations in TNBC [47]. However, this is the first report to delineate oncogenic interactions and cooperation between genes containing germline and somatic mutations in TNBC and non-TNBC and to investigate the differences in mutation burden between the two types of breast cancer. Here, we summarize the innovative aspects and clinical significance of the results from this investigation.

### 4.1. Discovery of Differentially Expressed and Differentially Mutated Gene Signatures

The discovery of highly significantly differentially somatic mutated gene signatures between TNBC and non-TNBC suggests that breast cancer may be amenable to mutation-based classification [48]. These results are consistent with our previous results on prostate cancer [49]. Given that somatic mutations drive tumorigenesis, this approach is likely to complement and further improve on traditional breast cancer classification based on transcription profiling [13]. Although our study is a cross-sectional study, our approach could also be useful to longitudinal studies for comparing mutation burden in early-stage versus late-stage tumors to identify genes that carry significantly higher mutation rates in the late stage compared to the early-stage subgroup of patients [48].

### 4.2. Germline and Somatic Mutated Gene Signatures

The discovery of functionally related genes containing both germline and somatic mutations is of particular interest. The clinical significance of this finding is that it provides a rational basis that breast cancer may be amenable to predictive modelling to identify patients at high risk of developing aggressive disease such as TNBC, a key step in the realization of precision prevention strategies. This discovery may also provide insights about how and when the cancer cells are likely to gain the propensity to acquire malignancy transformation into a lethal disease.

### 4.3. Gene Regulatory Networks and Signalling Pathways Enriched for Mutations

The discovery of gene regulatory networks and signalling pathways enriched for germline and somatic mutations is highly significant. It suggests that breast cancer is an emergent property of molecular networks and signalling pathways enriched for germline and somatic mutations. The investigation further revealed that interaction and cooperation between germline and somatic mutations during tumorigenesis occurs through gene regulatory networks and signalling pathways. The clinical significance of these findings is that such signalling pathways could be used as therapeutic targets.

### 4.4. Integrating Germline with Somatic Mutations

The majority of the germline mutations discovered thus far through GWAS map to noncoding regions such as intronic regions with undefined functions and their causal relationship with the disease have not been characterized. This investigation demonstrates that integrating germline with somatic mutation information provides a rational basis for establishing causal relationship between germline mutations and tumorigenesis. This is important given the limited evidence showing that cancer susceptibility variants are preferential targets for somatic mutations [50] and the discovery of germline and somatic mutations in oncogenes [51]. Although some germline and somatic mutations reported here could be passenger mutations, they bear the imprints of the mechanisms that generated them which have not been masked by the process of natural selection [51] and thus provide insights into the aetiologies, pathogenesis, and clonal evolutionally process of TNBC and non-TNBC [27].

As noted earlier in this report, to date, genetic variants are being incorporated in risky prediction models such as polygenic risk scores [25, 26]. These risk prediction models have modest success in risk assessment and currently are of limited practical use [25, 26]. One way to overcome the limitations of these risk prediction models and to improve their potential for clinical utility may be leveraging polygenic risk scores by integrating germline with somatic mutation information using gene expression data as the intermediate phenotype as demonstrated here to develop more robust risk prediction models. Although we did not address integration of polygenic scores with gene expression data in this study, many of the genetic variants used in this study have been used in the development and validation of polygenic risk score models in breast cancer [25, 26]. Moreover, a recent study showed that integrating polygenic risk scores with gene expression data is a powerful approach to unravelling complex traits [52] suggesting that such an approach is feasible.

In this study, we used the PAM50 and MammaPrint clinically validated and FDA-approved prognostic assays [15–19] to validate and test the ability of mutated genes discovered in this study to function as potential clinically actionable biomarkers. Apart from revealing the presence of many mutated genes from this study in those assays, the results of the study suggest that germline and somatic mutated genes could be incorporated in current genetic screening tests for stratifying patients and identifying patients at high risk of developing TNBC and non-TNBC [53, 54]. Given that germline mutated genes have far-ranging pathway-dependent influence on the somatic landscape as demonstrated here and in previous studies [55, 56], they could serve as early determinants of acquired somatic changes driving tumorigenesis. Taken together, the results of this investigation show that integration of germline with somatic mutation information has the promise of facilitating the realization of precision prevention in breast cancer.

### 4.5. Limitations

This study delineated the germline-somatic mutation interaction landscape in TNBC and non-TNBC. However, limitations must be acknowledged. Both GWAS and TCGA data sets lack diversity in ethnic population and clinical phenotype representation that would further inform these results. This limited progress must be balanced against the recognition that GWAS and TCGA studies have almost been exclusively focused on women of European ancestry. There is need for similar studies including women from underrepresented ethnic populations to ensure equitable use of genomic information to improve human health and eliminate health disparities [57]. We did not investigate the impact of mutations on gene function, gene expression, response to therapy, and survival, in part, because of the lack of specificity of the mutation information used. Notwithstanding this limitation, the impact of germline and somatic mutations on response to therapy and survival has been reported in TNBC [58]. Moreover, previous studies by our group and others have shown that germline and somatic mutations disrupt splice sites, binding sites, and gene regulatory elements such as enhancers [59, 60]. Another important limitation is that we did not extend the study to investigate subtypes in each type of breast as information on clinical subtyping was not available for both GWAS and genomic data, making such an undertaking beyond the scope of this study. Overall, despite some limitations which we readily acknowledge, and many of which are beyond the scope of this study, the results of this investigation suggest that in the context of breast cancer, TNBC and non-TNBC can be considered as emergent properties of molecular networks and signalling pathways influenced by alterations in the germline and somatic genomes acting cooperatively to drive and shape the clinical phenotypes. Finally, the majority of germline mutations used here are not breast cancer type-specific, a limitation emanating from the design nature of GWAS focused on cases and controls rather than types of breast cancer, which is beyond the scope of this investigation.

## 5. Conclusions

The investigation revealed oncogenic interactions and cooperation between genes containing germline and somatic mutations and showed that these complex arrays of interacting genetic factors occur through molecular networks and signalling pathways driving TNBC and non-TNBC. The investigation revealed differences in gene expression and somatic mutation burden between TNBC and non-TNBC. Further research is recommended to validate and ascertain the specificity of germline mutations to TNBC and non-TNBC in different ethnic populations including African American women to ensure equitable use of genomic information to improve human health.

## Figures and Tables

**Table 1:** List of 30 significantly differentially expressed genes mutated in TNBC and non-TNBC with high somatic mutation events per gene.

Genes	Chromosome position	Adjusted *P* value	TNBC somatic mutation events	Non-TNBC somatic mutation events
*COPE*	19p13.11	4.05*E* − 02	3	
*ENPP5*	6p21.1	6.09*E* − 17	3	
*RBM22*	5q33.1	1.44*E* − 18	3	
*AGPAT9*	4q21.23	4.45*E* − 13	2	
*ALB*	4q13.3	1.47*E* − 05	2	
*ASF1B*	19p13.12	1.65*E* − 14	2	
*BMP4*	14q22.2	5.59*E* − 29	2	
*C3orf62*	3p21.31	3.01*E* − 09	2	
*CALB2*	16q22.2	3.60*E* − 24	2	
*CAP1*	1p34.2	2.88*E* − 04	2	
*CRIP1*	14q32.33	4.35*E* − 17	2	
*FANCF*	11p14.3	1.78*E* − 06	2	
*FGD1*	Xp11.22	1.18*E* − 14	2	
*FHL3*	1p34.3	2.20*E* − 20	2	
*FKBPL*	6p21.32	2.11*E* − 11	2	
*GATA3*	10p14	5.11*E* − 133		99
*FOXA1*	14q21.1	3.57*E* − 144		23
*FRMPD4*	Xp22.2	3.50*E* − 07		21
*WNK3*	Xp11.22	8.42*E* − 20		18
*PHKA2*	Xp22.13	4.75*E* − 02		17
*ACACB*	12q24.11	7.79*E* − 15		16
*NRCAM*	7q31.1	4.13*E* − 04		16
*RAB3GAP2*	1q41	2.57*E* − 02		16
*SETX*	9q34.13	3.75*E* − 04		16
*CBLB*	3q13.11	1.10*E* − 02		15
*COL4A6*	Xq22.3	7.99*E* − 15		15
*DOCK3*	3p21.2	6.65*E* − 18		15
*ABCB11*	2q31.1	4.93*E* − 02		14
*CDH8*	16q21	4.82*E* − 03		14
*CNTNAP3B*	9p11.2	1.64*E* − 06		14

Note: blank cells in the 4^th^ and 5^th^ columns indicate that the gene is not mutated in that type of breast cancer.

**Table:** (a) Top 30 genes containing both germline and somatic mutations among genes significantly associated with TNBC

Genes	Chromosome position	Genetic variant	GWAS *P* value	Expression *P* value	Mutation events
*CREBBP*	16p13.3	rs12920416	8.00*E* − 07	3.83*E* − 06	7
*ARID1B*	6q25.3	rs140842923	3.00*E* − 06	7.25*E* − 05	6
*BRCA1*	17q21.31	rs1799950	2.00*E* − 04	3.95*E* − 07	5
*ERBB4*	2q34	rs13393577	9.00*E* − 14	9.03*E* − 41	5
*FHOD3*	18q12	rs9956546	2.90*E* − 06	1.62*E* − 19	5
*TNRC6B*	22q13.1	rs12483853	1.00*E* − 18	9.59*E* − 06	5
*ARHGAP24*	4q21.23	rs71599425	6.00*E* − 06	4.05*E* − 44	4
*ARHGAP5*	14q12	rs140783387	3.00*E* − 07	6.22*E* − 15	4
*CNTNAP2*	7q35	rs10487920	3.90*E* − 04	2.35*E* − 05	4
*DMD*	Xp21.1	rs1293906	9.00*E* − 06	2.42*E* − 40	4
*EFR3B*	2p23.3	rs1971136	5.00*E* − 09	4.51*E* − 04	4
*KIAA0907*	1q22	rs11406084	7.00*E* − 06	4.50*E* − 16	4
*MSH3*	5q11-q12	rs6151904	1.24*E* − 02	2.45*E* − 30	4
*MYO10*	5p15.1-p14.3	rs2562343	9.20*E* − 03	1.56*E* − 25	4
*MYT1*	20q13.33	rs6062356	3.00*E* − 06	5.60*E* − 03	4
*RELN*	7q22	rs17157903	5.00*E* − 02	8.36*E* − 21	4
*SPAG17*	1p12	rs1962373	1.00*E* − 06	7.67*E* − 05	4
*TRIM46*	1q22	rs4971059	5.00*E* − 11	1.14*E* − 17	4
*ZFPM2*	8q23.1	rs12546444	8.00*E* − 11	3.46*E* − 20	4
*ADCY9*	16p13.3	rs11076805	1.00*E* − 08	1.31*E* − 25	3
*AKAP9*	7q21.2	rs10644111	3.00*E* − 11	2.70*E* − 06	3
*ASH1L*	1q22	rs10796944	7.00*E* − 10	5.39*E* − 06	3
*ASXL2*	2p23.3	rs144079028	9.00*E* − 06	1.16*E* − 04	3
*ATM*	11q22-q23	rs1801516	2.00*E* − 04	1.35*E* − 08	3
*ATXN1*	6p22.3	rs3819405	2.00*E* − 08	3.92*E* − 06	3
*BAHCC1*	17q25.3	rs8074440	3.00*E* − 06	2.80*E* − 02	3
*CASZ1*	1p36.22	rs199867187	1.00*E* − 06	6.83*E* − 04	3
*CHST9*	18q11.2	rs1436904	1.00*E* − 14	1.52*E* − 11	3
*CNTNAP1*	17q21.2	rs72826962	5.00*E* − 09	8.69*E* − 09	3
*DNAH11*	7p15.3	rs7971	2.00*E* − 08	3.32*E* − 08	3

**Table:** (b) Top 30 genes containing germline and somatic mutations significantly associated with non-TNBC

Genes	Chromosome position	Genetic variant	GWAS *P* value	Expression *P* value	Mutation events
*DMD*	Xp21.1	rs1293906	9.00*E* − 06	5.33*E* − 107	41
*NOTCH2*	1p12	rs372562666	6.00*E* − 27	1.22*E* − 07	27
*RELN*	7q22	rs17157903	*P* < 0.05	2.13*E* − 56	22
*ATM*	11q22-q23	rs1801516	2.00*E* − 04	6.05*E* − 12	21
*RB1*	13q14.2	rs2854344	7.00*E* − 03	9.52*E* − 04	20
*ERBB2*	6p21.3	rs1801201	2.00*E* − 02	6.43*E* − 13	19
*ASH1L*	1q22	rs10796944	7.00*E* − 10	4.72*E* − 02	18
*MADD*	11p11.2	rs11039183	6.00*E* − 06	5.56*E* − 03	18
*ITPR1*	3p26.1	rs6787391	9.00*E* − 19	8.69*E* − 15	17
*FAM208B*	10p15.1	rs55910451	4.00*E* − 07	6.02*E* − 06	16
*ABCA8*	17q24.2	rs36059695	6.00*E* − 08	1.22*E* − 56	15
*ASXL2*	2p23.3	rs144079028	9.00*E* − 06	1.01*E* − 05	15
*CNTNAP2*	7q35	rs10487920	3.90*E* − 04	1.58*E* − 25	15
*GRIN3A*	9q31.1	rs10512287	2.30*E* − 04	1.08*E* − 10	15
*PIK3R1*	5q13.1	rs184886	2.00*E* − 06	2.22*E* − 43	15
*TNRC6B*	22q13.1	rs12483853	1.00*E* − 18	1.78*E* − 13	15
*CASZ1*	1p36.22	rs199867187	1.00*E* − 06	9.91*E* − 15	14
*FRMD4A*	10p13	rs10906522	1.00*E* − 07	7.13*E* − 36	14
*KCNH7*	2q24.3	rs148760487	2.00*E* − 08	1.33*E* − 02	14
*TACC2*	10q26.13	rs2253762	2.00*E* − 09	1.68*E* − 06	14
*ABCC4*	13q32.1	rs1926657	2.00*E* − 06	4.34*E* − 02	13
*ADCY9*	16p13.3	rs11076805	1.00*E* − 08	2.00*E* − 04	13
*CCDC88C*	14q32.11	rs941764	8.00*E* − 13	8.06*E* − 18	13
*FGFR2*	10q26.13	rs35054928	2.00*E* − 322	8.49*E* − 05	13
*SPTBN2*	11q13.2	rs55908905	8.00*E* − 06	6.77*E* − 03	13
*ATR*	3q23	rs1802904	2.24*E* − 02	1.70*E* − 02	12
*BRCA1*	17q21.31	rs1799950	2.00*E* − 04	6.38*E* − 14	12
*BRCA2*	13q13.1	rs11571833	3.00*E* − 15	5.15*E* − 27	12
*CASP8*	2q33.1	rs3769821	4.00*E* − 18	4.83*E* − 02	12
*CDH12*	5p14.3	rs66783663	5.00*E* − 06	1.57*E* − 76	12

**Table:** (c) Top 30 genes with both germline and somatic mutations distinguishing TNBC from non-TNBC

Gene name	Chromosome position	SNP_ID	GWAS *P* value	Expression *P* value	GWAS event	TNBC mutation event	Non-TNBC mutation event
*AGPAT9*	4q21.23	rs1963045	2.00*E* − 06	4.45*E* − 13	1	2	
*FKBPL*	6p21.32	rs169494	3.10*E* − 08	2.11*E* − 11	1	2	
*IGSF21*	1p36.13	rs2992756	2.00*E* − 15	3.82*E* − 10	1	2	
*BABAM1*	19p13.11	rs8170	7.00*E* − 21	2.68*E* − 06	1	1	
*MCM8*	20p12.3	rs16991615	2.00*E* − 09	2.25*E* − 11	1	1	
*MYC*	8q24.21	rs11780156	1.00*E* − 13	2.01*E* − 15	1	1	
*NFIX*	19p13.13	rs78269692	2.00*E* − 09	1.79*E* − 14	1	1	
*FRMD4A*	10p13	rs10906522	1.00*E* − 07	2.88*E* − 24	1		14
*KCNH7*	2q24.2	rs148760487	2.00*E* − 08	3.06*E* − 02	1		14
*ABCC4*	13q32.1	rs1926657	2.00*E* − 06	1.12*E* − 15	1		13
*CCDC88C*	14q32.11	rs941764	8.00*E* − 13	1.97*E* − 07	1		13
*CDH12*	5p14.3	rs66783663	5.00*E* − 06	5.53*E* − 05	1		12
*MAGI3*	1p13.2	rs1230666	4.00*E* − 10	1.98*E* − 17	1		12
*TNS1*	2q35	rs6436017	3.00*E* − 10	2.83*E* − 08	1		11
*BLM*	15q26.1	rs8037430	1.00*E* − 03	3.35*E* − 39	2		10
*DNM3*	1q24.3	rs1894633	2.00*E* − 06	6.20*E* − 04	1		10
*L3MBTL3*	6q23.1	rs6569648	3.00*E* − 12	2.41*E* − 09	1		10
*BRIP1*	17q23.2	Deletion	2.00*E* − 03	2.15*E* − 08	2		9
*FOXP1*	3p13	rs6805189	5.00*E* − 08	3.76*E* − 50	1		9
*ABCF1*	6p21.33	rs3132610	1.00*E* − 06	8.60*E* − 31	1		8
*ADCY3*	2p23.3	rs6725517	3.00*E* − 12	7.56*E* − 20	1		8
*COL1A1*	17q21.33	rs2075555	8.00*E* − 08	8.64*E* − 07	1		8
*CUX1*	7q22.1	rs71559437	5.00*E* − 12	3.21*E* − 04	1		8
*EWSR1*	22q12.2	rs132390	3.00*E* − 09	1.25*E* − 07	1		8
*EXOC3*	5p15.33	rs190811224	5.00*E* − 06	9.27*E* − 03	1		8
*IGF1R*	15q26.3	rs1546713	3.00*E* − 02	7.77*E* − 33	2		8

## Data Availability

GWAS data is provided in Supplementary Table SG provided as supplementary materials to this report. Additional GWAS information is available at the GWAS catalogue managed by the European Bioinformatics Institute: https://www.ebi.ac.uk/gwas/. Original gene expression and mutation data are available at the TCGA via the Genomics Data. Additional data on mutated and nonmutated genes associated with and distinguishing the two diseases is provided in the supplementary tables in this report.

## Supplementary Materials

Supplementary Materials All additional results from the analysis are shown in the supplementary tables described. Supplementary Table SG: GWAS information-genetic variants and genes associated with an increased risk of developing breast cancer. Supplementary Table SM: somatic mutated genes significantly associated with TNBC vs. control and non-TNBC vs. control. Supplementary Table SN: nonsomatic mutated genes significantly associated with TNBC vs. control and non-TNBC vs. control. Supplementary Table S2A1: list of all somatic mutated genes significantly associated with TNBC. Supplementary Table S2A2: list of all somatic mutated genes significantly associated with non-TNBC. Supplementary Table S1: differentially expressed and uniquely somatic mutated genes between TNBC and non-TNBC. Supplementary Table SA3: significantly differentially expressed germline mutated genes with and without somatic mutations in TNBC. Supplementary Table SB3: significantly differentially expressed germline mutated genes with and without somatic mutations in non-TNBC. Supplementary Table S3A: genes containing both germline and somatic mutations significantly associated with TNBC. Supplementary Table S3B: genes containing both germline and somatic mutations significantly associated with non-TNBC. Supplementary Table S3C: a complete list of 251 genes containing both germline and somatic mutations distinguishing TNBC from non-TNBC.
